# Cortical Activation to Social and Mechanical Stimuli in the Infant Brain

**DOI:** 10.3389/fnsys.2021.510030

**Published:** 2021-06-24

**Authors:** Marisa Biondi, Amy Hirshkowitz, Jacqueline Stotler, Teresa Wilcox

**Affiliations:** ^1^Tobii Pro, College Station, TX, United States; ^2^Department of Psychological & Brain Sciences, Texas A&M University, College Station, TX, United States; ^3^Baylor College of Medicine, Houston, TX, United States; ^4^Department of Psychology, Florida Atlantic University, Boca Raton, FL, United States

**Keywords:** infants, temporal cortex, fNIRS, mechanical stimuli, social stimuli

## Abstract

From the early days of life infants distinguish between social and non-social physical entities and have different expectations for the way these two entities should move and interact. At the same time, we know very little about the cortical systems that support this early emerging ability. The goal of the current research was to assess the extent to which infant’s processing of social and non-social physical entities is mediated by distinct information processing systems in the temporal cortex. Using a cross-sectional design, infants aged 6–9 months (Experiment 1) and 11–18 months (Experiment 2) were presented with two types of events: social interaction and mechanical interaction. In the social interaction event (patterned after Hamlin et al., 2007), an entity with googly eyes, hair tufts, and an implied goal of moving up the hill was either helped up, or pushed down, a hill through the actions of another social entity. In the mechanical interaction event, the googly eyes and hair tufts were replaced with vertical black dots and a hook and clasp, and the objects moved up or down the hill via mechanical interactions. FNIRS was used to measure activation from temporal cortex while infants viewed the test events. In both age groups, viewing social and mechanical interaction events elicited different patterns of activation in the right temporal cortex, although responses were more specialized in the older age group. Activation was not obtained in these areas when the objects moved in synchrony without interacting, suggesting that the causal nature of the interaction events may be responsible, in part, to the results obtained. This is one of the few fNIRS studies that has investigated age-related patterns of cortical activation and the first to provide insight into the functional development of networks specialized for processing of social and non-social physical entities engaged in interaction events.

## Introduction

One of the most important distinctions that infants must make to effectively navigate their world is between social and non-social physical entities. In a seminal paper, Premack (1990) proposed that infants possess distinct knowledge systems for perceiving, apprehending, and reasoning about social and non-social physical entities (for similar views see Gelman, 1990; Mandler, 1992, 2000; Leslie, 1994, 1995). According to this view, infants perceive objects that engage in self-propelled, goal-directed behavior as possessing psychological states and categorize these as social entities. In contrast, infants perceive objects that move only by an external force and in ways constrained by mechanical laws (rather than psychological states) as physical entities. Since Premack (1990), a large number of studies have been conducted to assess the extent to which the naïve human mind is sensitive to this distinction.

### Infants Processing of Social and Non-social Physical Entities

There is now a great deal of behavioral evidence to support the idea that from the early days of life infants both distinguish between social and non-social physical entities and have different expectations for the way these two types of entities should move and interact (for reviews see Spelke and Kinzler, 2007; Wynn, 2008; Baillargeon et al., 2011; Baillargeon and Carey, 2012; Buyukozer Dawkins et al., 2019). Even very young infants expect *physical objects* to move in accordance with basic mechanical laws and recognize when the outcomes of physical events are inconsistent with these laws (Spelke, 1994; Baillargeon, 1998). Expectations become more nuanced over time, as infants gain experience in the physical world (Baillargeon et al., 2011). For example, early in the first year infants expect inanimate physical objects to remain stationary unless there is a cause for displacement, such as being hit by another moving object. By the end of the first year, infants recognize the amount of displacement that occurs when a moving object hits a stationary object depends on a number of factors, such as the relative size of the “displacer” and the “displacee” (Kotovsky and Baillargeon, 1994, 1998, 2000; Wang et al., 2003).

In contrast, objects that display autonomous motion and engage in goal-directed behavior are viewed as *social entities.* From the first months of life infants appreciate that the actions of social entities are guided by mental states, such as motivation, intention, or volition (Wynn, 2008; Hamlin, 2013; Baillargeon et al., 2014; Buyukozer Dawkins et al., 2019). Infants’ assessment of social interactions also includes an evaluative component (Premack and Premack, 1997). For example, infants distinguish between cooperative and uncooperative behavior and show a preference for agents that engage in the former (Hamlin et al., 2007). Older infants come to appreciate the complexities of social behavior and consider factors such as the cost-benefit ratio of helping (Sommerville et al., 2018), whether entities are from the same social group (Pun et al., 2018), and prior experience with the entity (Hamlin and Wynn, 2012; Hamlin et al., 2013) when interpreting and evaluating the behavior of social agents. Although some researchers have suggested that these findings can be explained by lower level processes, such as perceptual differences between displays or a preference for positive valence (Scarf et al., 2012a,b), there is now an abundance of evidence from a number of different labs demonstrating an early emerging and enduring propensity to differentiate between cooperative and uncooperative behavior that guides infant’s attention to and behavior toward social agents (Holvoet et al., 2016; Margoni and Surian, 2018).

### Processing of Social and Non-social Physical Entities: Cortical Networks

Given evidence that even very young infants are sensitive to the distinction between social and non-social physical entities, one might wonder about the extent to which the immature human brain is prepared to differentially respond to interactions involving these two types of entities. First, a look at the mature brain is warranted.

Much of the early neuroimaging work focused on understanding the extent to which the mature brain represents the distinction between biological (human) versus non-biological (mechanical) motion. This work revealed the posterior superior temporal sulcus (pSTS) as critical to the analysis of biological human motion, showing greater sensitivity to point-light and animated displays containing upright human motion, as compared to displays containing scrambled, disjointed or inverted human motion, and as compared to displays containing robot or mechanical motion (Grossman et al., 2000, 2004, 2005; Grossman and Blake, 2001; Pelphrey et al., 2003; Peuskens et al., 2005). Typically, responses are more robust in the right than the left hemisphere. In contrast, movement of mechanical, robotic, or inanimate objects typically leads to activation in the middle temporal gyrus (MTG) (Beauchamp et al., 2002, 2003; Martin and Weisberg, 2003; Beauchamp and Martin, 2007; Han et al., 2013).

Many have questioned, however, how to best characterize the underlying nature of these results. Biological motion, particularly human motion, is inherently purposeful and goal directed. Hence, the pattern of activation observed might be better explained by the distinction between animate and inanimate, or that between social and mechanical interactions, than between biological and non-biological visual motion. To test this hypothesis, Martin and Weisberg (2003) presented adult participants with displays composed of geometric shapes that engaged in human-inspired social interactions (e.g., dancing, fishing, sharing, and playing) or mechanical interactions inspired by the movement of inanimate objects (e.g., pinball, cannon, conveyer belt, and crane). They found that viewing vignettes involving animated social interactions lead to activation in the pSTS (more robust in right than left hemisphere), whereas viewing vignettes in which objects with mechanical movements engage in automated actions (which have little or no perception of a human instigating the action) leads to greater activation in the MTG (more robust in left than right hemisphere). There is converging evidence, from other researchers, that right STS shows greater activation to displays containing animate than inanimate motion patterns (Wheatley et al., 2007), supporting the hypothesis that the presence of animate, intentional, goal-directed behavior is critical to pSTS activation.

Other studies have focused on adult’s processing of socially-relevant behavior observed within the context of more complex situations. For example, when adult participants are asked to reason about the extent to which an individual’s behavior is motivated by mental states (Saxe and Kanwisher, 2003; Saxe and Powell, 2006) or about the intentionality of harmful behaviors (Young et al., 2010; Koster-Hale et al., 2013), activation is obtained in the temporal parietal junction (TPJ) and is often right lateralized. Furthermore, activation patterns to social as compared to mechanical interactions are dissociated – these two types of interactions engage distinct cortical networks that involve different regions in temporal, parietal, and/or frontal cortex (Jack et al., 2013).

Surprisingly little is known about the origins and development of the cortical patterns of activation to physical and social entities reported in adult populations. In one of a limited number of studies, Lloyd-Fox et al. (2009; for a replication see van der Kant et al., 2018) investigated the extent to which the infant cortex responds differentially to social and mechanical stimuli using functional near-infrared spectroscopy (fNIRS). Infants aged 5 months saw a video of a woman engaged in socially-relevant actions (e.g., moved her hands to play peek-a-boo), and a video of inanimate objects undergoing mechanical movements (e.g., machine cogs, pistons, or a moving mechanical toy), on alternating test trials. Bilateral activation (relative to baseline stimuli, which were static pictures of transport vehicles) was obtained in posterior areas of the superior temporal region in response to the dynamic social stimuli. The dynamic mechanical stimuli also elicited activation in posterior areas of the superior temporal region, but this response was observed only in the right hemisphere and was significantly less robust than that observed in response to the social stimuli.

Biondi et al. (2016) investigated cortical responses to action sequences performed by human and mechanical hands also using fNIRS. In this study, infants aged 6–10 months viewed events in which a human hand or a mechanical hand (grabber device) engaged in functionally relevant actions (e.g., used a tool to pound a nail or to scoop and pour salt). Other infants viewed control events that were identical to the experimental events except that the action patterns performed by the human or the mechanical hand were not functionally relevant (e.g., the hand engaged in pounding motions or scooping/pouring motions, without coming in contact with the nail or the salt). Anterior areas of the right superior temporal region showed greater activation to the human than the mechanical hand regardless of whether the event was functionally relevant. In contrast, in analogous areas in the left temporal cortex greater activation was obtained to the human than the mechanical hand but only when the hand engaged in actions that were functionally relevant. The dissociation between hand and event reveals that object function is processed differently when produced by a human (social) agent than a mechanical (non-social) agent, which is directly relevant to the present research.

Finally, Grossmann et al. (2013) assessed hemodynamic responses in motor and nearby temporal cortex to human and robotic motion in 4-month-olds. Infants saw events in which the form of an object (human or robot) was crossed with the motion that the object displayed (human or robot). Two main findings emerged: (1) areas in the right premotor cortex responded selectively to robot as compared to human motion (regardless of whether the motion was seen on a human or robot form), and (2) left temporal cortex responded selectively to congruent (human–human/robot–robot) as compared to incongruent (human–robot/robot–human) form-motion pairings. Unlike Lloyd-Fox et al. (2009) and Biondi et al. (2016), actions of human and non-human entities did not elicit distinct patterns of activation in temporal cortex. This may be due, at least in part, to the fact that they measured activation at temporal areas more anterior and superior to those of Lloyd-Fox et al. (2009) and Biondi et al. (2016).

In summary, there are data to suggest that social and mechanical stimuli elicit distinct patterns of activation in the naïve, infant cortex. At the same time, we are limited in what we know about why such patterns are observed and the conditions under which these differences emerge. The current research will fill this gap in knowledge by systematically assessing infants’ cortical responses to interactions in which social and mechanical entities are engaged. The focus here was to better understand the extent to which the nature of the interactions influences patterns of cortical activation.

### Current Research

The goal of the current research was to assess specialization of cortical activation in response to viewing social and non-social physical entities engaged in social and mechanical interactions, respectively. If the processing of social and mechanical interactions is mediated by distinct information processing systems, we would expect different patterns of cortical activation to these two types of events. We also sought to examine the extent to which patterns of cortical activation change during the first 18 months of life. Since there are behavioral changes in response to social and mechanical entities over the first 2 years of life, as well as age-related changes in neural systems recruited for object processing (Wilcox and Biondi, 2015a,b), we anticipated finding different patterns of cortical activation between younger and older infant age groups. Hence, infants aged 6–9 months (Experiment 1) and 11–18 months (Experiment 2) were tested cross-sectionally.

## Experiment 1

**FIGURE 1:**
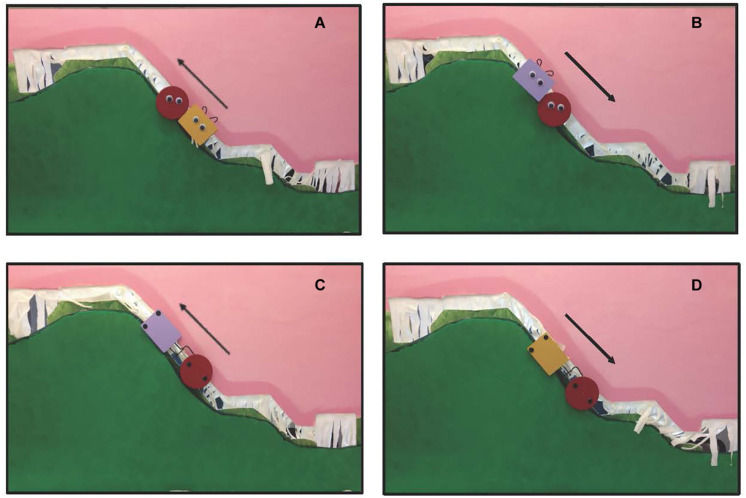
**(A)** A social help event; **(B)** a social hinder event; **(C)** a mechanical pull event; **(D)** a mechanical push event. Arrows indicate direction of movement. Social events were patterned after Hamlin et al. (2007).

Infants aged 6–9 months were tested in one of two conditions: experimental or control. Infants in the *experimental condition* saw social and mechanical interaction events (Figures 1A–D). In the social interaction event, patterned after Hamlin et al. (2007), an entity with googly eyes, hair tufts, and an implied goal of moving up the hill was either helped up, or pushed down, a hill through the actions of another entity with googly eyes and hair tufts. In the mechanical interaction event, the googly eyes and hair tufts were replaced with vertical black dots and a hook or clasp, and the objects moved up or down the hill via mechanical interactions. The colors and shapes of the objects, and the trajectories that they traveled, were identical across the events. Hence, the primary difference between the two event types was whether the features that the entities possessed, and the interactions in which they engaged, were social or mechanical in nature.

To assess the extent to which the cortical responses obtained are specific to the nature of the interactions in which the objects engaged, another group of infants were tested in a *control condition.* A similar protocol was used except the two entities in the events never physically contacted each other (they remained at a distance of about 4 cm). In the control social events, the social entities moved up or down the hill in synchrony, but never came in contact. Likewise, in the control mechanical events, the mechanical entities moved up and down the hill in synchrony, without making contact.

Hemodynamic responses were measured in the temporal cortex while infants viewed the events. We started with this age group because it is similar to the age group tested by Biondi et al. (2016) using the same headgear configuration. This is also similar to the age group tested in behavioral tasks by Hamlin et al. (2007).

### Method

#### Participants

Infants aged 6–9 months participated [*N* = 36; 13 females; age in days *M* = 240.5 (*SD* = 45.1); range = 190–288 days]. Nineteen additional infants were tested but eliminated from the sample due to infant removing the headgear (*n* = 2), equipment failure (*n* = 1), fussiness or crying (*n* = 7), or difficulty obtaining an optical signal (*n* = 9). The percentage of infants excluded here is typical for fNIRS studies with infant populations. Infants were pseudo-randomly assigned to one of two conditions: experimental (*n* = 18) or control (*n* = 18).

Parents reported their infant’s ethnicity as Hispanic (*n* = 7), non-Hispanic (*n* = 28), or unknown/not reported (*n* = 1). Additionally, parents reported their infant’s race as White/Caucasian (*n* = 27), Black/African American (*n* = 1), or multiple races (*n* = 8). In this and the next experiment, parents were recruited primarily by social media and commercially available lists and given $5 or a lab t-shirt for their participation. The experimental procedure was explained to the parents and informed consent was obtained prior to testing.

#### Task and Procedure

Infants were randomly assigned to either an experimental or a control condition. Infants in the *experimental condition* were presented with two event types in a blocked design: social and mechanical (Figure 1, see Supplementary Videos). Trials were 12 s in length and began with the agent (defined as an entity with active power or cause) and patient (defined as the entity affected by, or on the receiving end of, an agent’s actions) entering the apparatus (1 s) and ended with the agent and patient exiting the apparatus (1 s). The remaining 10 s are as described. In the *experimental social event* infants saw the patient, a round object with animate properties, attempt to move up the steep slope of hill and slide backward, twice. Next, the agent, a second object of a different shape and color, also with animate properties, either approached the patient from the bottom of the hill and helped it move to the top of the hill, *help event*, or approached the patient from the top of the hill and pushed it to the bottom of the hill, *hinder event*. Hamlin et al. (2007) report that as soon as infants are able to physically reach for objects, about 5 months of age, they show a preference for a prosocial as compared to antisocial agent in a subsequent choice task. In the *experimental mechanical event*, infants were tested using the same protocol except that (a) the googly eyes were replaced with black circles of approximately the same size that were positioned vertically, rather than horizontally, on the shape, (b) the curly-hair wires were shaped like a hook (in the case of the agent) or a clasp (in the case of the patient) and located on the side of the object, and (c) the patient was either pulled up the hill or pushed down the hill by means of a mechanical interaction. In the *pull event*, the patient (red circle) sat at the bottom of the hill with an agent (of a different shape and color) on its left. The agent attached itself to the patient via the hook and clasp and moved steadily to the top of the hill. In the *push event*, the patient sat at the top of the hill with an agent on its left. The agent attached itself to the patient via the hook clasp and moved steadily to the bottom of the hill. Because of differences in how the social and mechanical events were produced we could not equate, across social and mechanical events, whether the agent started to the left or right of the patient. We did equate whether the direction of movement was up or down the hill.

Each infant was presented with three pairs of social (help/hinder) and three pairs of mechanical (push/pull) trials for a total of 12 trials in a blocked design. The order in which infants saw the two blocks was randomly determined. Within each block, infants saw helper/hinderer events (social block) and push/pull events (mechanical block) on alternating trials. Whether infants saw the helper (or hinderer) event first, or the push (or pull) event first, within each block was randomly determined.

**FIGURE 2:**
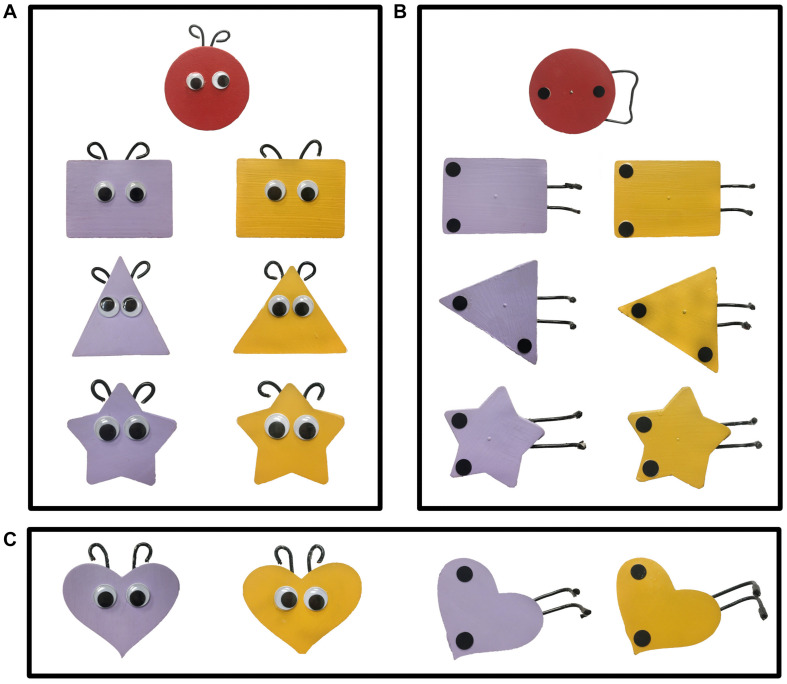
The target and three pairs of objects seen in the three pairs of **(A)** social events and **(B)** mechanical events of Experiments 1 and 2. The fourth pair of objects **(C)** were used in the choice task of Experiment 1.

A different pair of objects (agents) was used for each of the three pairs of trials within each block (Figure 2); the two objects of each pair differed only on color (yellow and purple). The agents, and the red patient, were all 9 cm at their widest point and moved by a stick from the back of the apparatus. Within each participant’s design, the same-colored object from each pair performed the same action (e.g., if the yellow object of the pair was a helper and the purple a hinderer, this held true for each of the three pairs of social events). However, the relation between color and direction differed across blocks (e.g., if in the social block the yellow object helped the patient up the hill, then in the mechanical block, the yellow object pushed the patient down the hill). Whether infants saw the yellow agent as the helper or hinderer was randomly assigned, and this assignment influenced the role of the remaining agents during all trial blocks.

Infants in the *control condition* were tested using the same protocol except that the two entities in each event moved synchronously but never came in contact; they were spatially separated by approximately 10 cm throughout the trial.

Infants sat in a Bumbo^®^, an infant booster seat, or their parents lap in a dark, quiet room and watched the events presented in a puppet-stage apparatus. Infants sat approximately 78 cm from the objects on the puppet stage. Two naive observers monitored infants’ looking behavior, via game controllers, through peepholes in muslin-covered wooden frames attached to the sides of the apparatus. Inter-observer agreement was calculated and averaged 95% (per trial and infant). Each trial was preceded, and followed, by a 10 s baseline period. During baseline a 23 cm square silver object, with yellow and blue pinwheel design, moved right (5 s) and the left (5 s) across the stage of the apparatus.

Total duration of looking (i.e., cumulative looking) to each trial was obtained. Trials where infants looked <5 s were excluded from analysis. This ensured that infants were attending to the event when hemodynamic responses were being measured.

#### Instrumentation

The imaging equipment was a TechEn CW7 (TechEn, Inc.) which contained eight fiber optic cables that delivered near-infrared light to the scalp of the participant (emitters), eight fiber optic cables that detected the diffusely reflected light at the scalp (detectors), and a control box that served as the source of the near-infrared light and the receiver of the reflected light. The control box produced light at wavelengths of 690 nm, which is more sensitive to deoxygenated blood (HbR), and 830 nm, which is more sensitive to oxygenated blood (HbO), with two laser-emitting diodes.

Laser power emitted from the end of the diode was 4 mW. Light was square wave modulated at audio frequencies of approximately 4–12 kHz. Each laser had a unique frequency so that synchronous detection could uniquely identify each laser source from the photodetector signal. Ambient illumination from the testing room did not interfere with the laser signals because environmental light sources modulate at a different frequency. However, for additional protection a light-blocking black cap was placed over the headgear. Fiber optic cables were 1 mm in diameter and 5 m in length. Each emitter delivered both wavelengths of light and each detector responded to both wavelengths. The raw signals were acquired at 50 Hz, were received by an electronic control box, and then relayed to a DELL desktop computer via a custom designed program (available from TechEn, Inc.).

**FIGURE 3:**
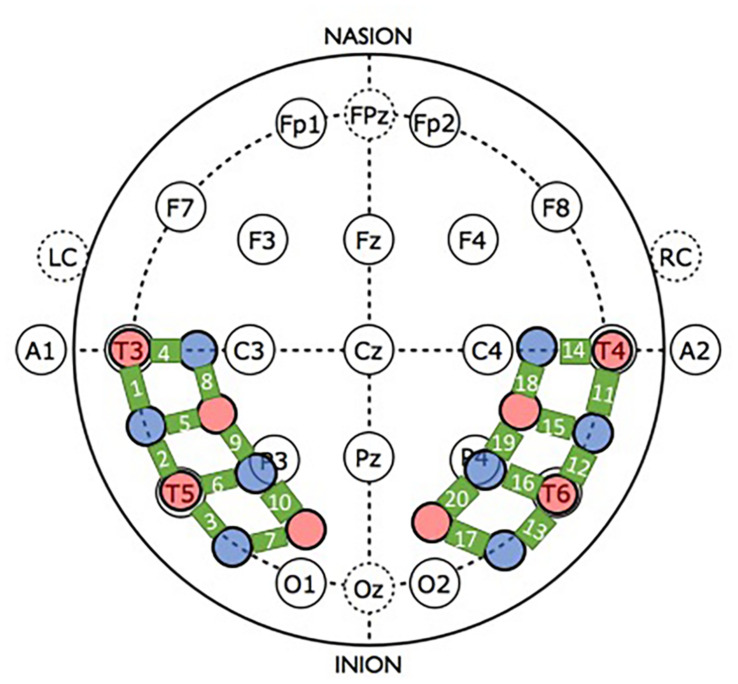
Headgear configuration and placement. The headgear consisted of two pads, placed over temporal and temporal-occipital regions of the left and right hemisphere, respectively. Four emitters (red circles) and four detectors (blue circles) were embedded in each pad. The bold green lines connecting the sources and detectors are channels, which are numbered. For placement of the headgear on the infant’s head, the left and right pads were anchored at T3 and T4, respectively, of the 10–20 International EEG system. Secondary anchors were O1 and O2. Emitter-detector distances were all 2 cm.

Prior to the experimental session, infants were fitted with a custom-made headgear, which secured the optodes to the scalp. Configuration of the sources and detectors within the headgear, location of corresponding channels, and placement of the headgear on the infant’s head in relation to the 10–20 International EEG system are illustrated in Figure 3. The pads in which the sources and detectors were embedded were rigid so the distance between the sources and detectors within each pad remained fixed at 2 cm; however, the bands connecting the two pads were elastic. Mean head circumference did not differ significantly by condition (experimental *M* = 44.7, *SD* = 1.73 and control *M* = 45.6, *SD* = 1.81), *t* = −1.46, *df* = 34, *p* = 0.154. Although the head circumference of the infants tested ranged from 41.5 to 49.0 cm, the difference in the amount of skull covered by the left and right segments of the headgear, each, differed by 1.69 cm between the smallest and largest head circumference, which is less than the source-detector distance.

For reliable placement of the headgear, prior to the experimental session infants’ heads were measured (circumference, nasion-inion, and IAC-IAC) and locations T3/T4 and O1/O2 were marked on the scalp with washable marker. Then, the headgear was placed on the infant’s head using T3/T4 as primary anchors (i.e., the most anterior and inferior sources in left and right hemispheres, respectively, were placed at T3 and T4) and O1/O2 as secondary anchors.

#### Processing of fNIRS Data

The fNIRS data were processed for each channel, trial, and condition separately. Processing was conducted using Matlab (Mathworks, Natick, MA, United States) HOMER2 v2.1^1^. The raw intensity data were converted to optical density units. Channels that demonstrated very low or very high optical density were excluded (i.e., if a channel’s mean density divided by the standard deviation of density was greater than SNR threshold of 2 the channel was pruned using enPruneChannels). A principal components analysis was then used to remove systemic physiology and other noise common across channels. As suggested by Wilcox et al. (2005) components that contributed 80% or more of the variance of the data were removed (enPCAFilter nSV set to 0.80). Data objectively categorized as containing motion artifacts (i.e., a change in filtered intensity greater than 5% in 1/20 s during the 2 s baseline and test event) were excluded (using hmrMotionArtifact). Following this, a bandpass filter (0.01–0.5 Hz) was applied to remove any low-frequency drift and remaining high-frequency noise (hmrBandpassFilt hpf set to 0.01 and lpf set to 0.50) and the pathway differential factor was set at 6 for both HbO and HbR. The data were then converted to relative concentrations of oxygenated (HbO) and deoxygenated (HbR) blood using the modified Beer-Lambert law. Finally, trials in which the infant looked less than 5 of the 12 s trial length were excluded from analysis.

In order to be included in the analysis, infants needed to complete at least 3 of the 6 trials for each event (social and mechanical). On the basis of the processing criteria described above, 72 of 432 possible trials (16.7%) were eliminated from analysis. The total percentage eliminated did not vary significantly between events within each condition: experimental social, 17 of 108 trials (17.6%) as compared to experimental mechanical, 20 of 108 trials (18.5%), *z*-score < 1.0; control social, 20 of 108 trials (18.5%) as compared to control mechanical, 15 of 108 trials (13.9%), *z*-score < 1.0.

Changes in HbO were examined 2 s prior to the onset of the test event, the 12 s test event, and the 10 s baseline. Changes in HbO, compared to baseline, were averaged over 7–12 s of each trial, then averaged over trials and participants to obtain a grand average for each group. The time epoch used for data analysis was chosen *a priori*. Viewing the first 5 s of the event gave infants the opportunity to identify whether the object was social or mechanical, and whether it moved on its own or there was a cause for motion. Allowing 2 s for the hemodynamic response to become initiated, changes in HbO should be detectable by 7 s and continue to the end of the trial.

Finally, in order to be included in the analysis, infants in this and the next experiment were required to contribute data from at least 13 of the total 20 channels, and at least 5 of 10 channels in a given hemisphere, for each of the two events (social and mechanical). We conducted a Little’s (1988) Missing Completely at Random (MCAR) test to assess whether the missing values were randomly distributed for each sample. Each test was not significant, suggesting the data are MCAR (experimental, mechanical: χ_2_ = 27.21, DF = 57, *p* = 1.00; experimental, social: χ_2_ = 40.04, DF = 57, *p* = 0.957; control, mechanical: χ_2_ = 178.00, DF = 191, *p* = 0.741; control, social: χ_2_ = 186.49, DF = 211, *p* = 0.887). The mean percentage of missing channels, averaged over condition, was 10.1%.

### Results

All frequentist statistics were conducted using IBM SPSS Statistics for Macintosh, Version 27.0 and all Bayesian statistics were conducted using JASP Version 0.13.1 for Macintosh.

#### Preliminary Analyses

Preliminary analysis of the looking time and optical imaging data revealed no significant main effects or interactions involving sex. Hence, this factor will not be included in the main analyses. However, given the relatively small number of males and females tested, null effects should be interpreted with caution.

#### Looking Time Data

Duration of looking time (in sec) was averaged across trials for each event and condition, separately. The looking times of the infants who viewed the experimental social (*M* = 11.1 s, *SD* = 0.80 s) and the control social (*M* = 11.3 s, *SD* = 1.13 s) events did not statistically differ, *t*(34) < 1. Likewise, the looking times of the infants who viewed the experimental mechanical (*M* = 10.8 s, *SD* = 0.90 s) and the control mechanical (*M* = 10.9 s, *SD* = 1.04 s) events did not statistically differ, *t*(34) < 1.

#### Optical Imaging Data

Our goal was to identify cortical regions of interest (ROIs) in response to the experimental social and mechanic events that were statistically robust. This approach has been used previously by a number of researchers (e.g., Lloyd-Fox et al., 2011; Biondi et al., 2016; Hirshkowitz et al., 2018). For each of the 20 channels (10 channels within each hemisphere) responses were averaged over 7–12 s. Responses were then averaged over trial and infant for each of the two events (social and mechanical), independently, to obtain a grand average. Given that HbO responses are typically more robust than HbR responses (Strangman et al., 2003), in this and the next experiment we focused our analyses on HbO. However, because HbR is important for identifying cortical activation (Obrig and Villringer, 2003) we also report HbR. Mean hemodynamic responses, including HbO and HbR, for Experiment 1 are reported in Supplementary Appendices A,B, respectively.

For each event, relative changes in the mean HbO for each channel were compared to 0 using one-sample *t*-tests. We had a directional hypothesis (changes in HbO would be in the positive direction), hence one-tailed tests were performed. The outcome of the one-sample *t*-tests, along with effect sizes using Cohen’s *d*, are reported in Supplementary Appendix A. In addition, Bayesian analyses were conducted to examine the robustness of the results (Kruschke, 2015). Bayesian analyses assess the extent to which the alternative hypothesis, an increase in HbO relative to 0 (the null hypothesis would be no increase in HbO relative to 0), was supported. A Bayes Factor (BF) indicates that the data obtained are *B* times more likely under the alternative than null hypothesis. For example, a BF of 3 indicates that the data are 3 times more likely under the alternative hypothesis. A BF between 1 and 3 indicates that there is weak evidence for the alternative hypotheses, between 3 and 20 indicates positive evidence, between 20 and 100 strong evidence, and >100 very strong evidence (Raftery, 1995). Bayes factors are also reported in Supplementary Appendix A. To be consider activated a channel had to meet three criteria: *p* < 0.05, *d* > 5.0, and BF > 3. The use of effect sizes and Bayesian factors, in addition to *p-*values, guards against Type 2 error, enhances the robustness and replicability of our results, and negates the need for adjustments for multiple planned comparisons, which are notoriously conservative (Gelman et al., 2012; Kruschke, 2015; Etz and Vandekerckhove, 2017). Finally, for ease of analysis, when two or more neighboring channels were statistically significant (*p* < 0.05, *d* > 5.0, and BF > 3) for at least one of the two experimental event types (social or mechanical) an ROI was computed by averaging responses across the activated channels. The statistical likelihood of two or more spatially adjacent (neighboring) channels producing false positive results is low (*p* = 0.016; see Lloyd-Fox et al., 2011; Biondi et al., 2016) providing further evidence as to the robustness of the results.

**FIGURE 4:**
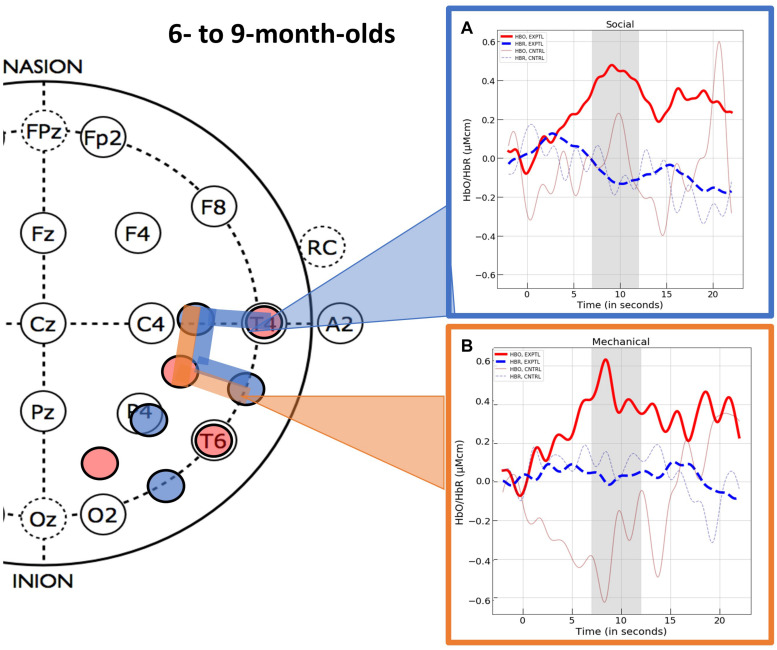
The hemodynamic response curves obtained in the ROIs identified in Experiment 1 with the 6- to 9-month-olds in the **(A)** social condition (the blue overlays) and the **(B)** mechanical condition (the orange overlays). The time course of the curves (x-axis) includes 2 s prior to the test event, the 12 s test trial (highlighted gray is the 5 s time epoch over which we averaged), and the 10 s baseline event. Units on the y-axis are moles.

This procedure revealed three spatially contiguous channels in the superior temporal region of the right hemisphere that were activated in response to the experimental social event (channels 14, 15, and 18) and two that were activated in response to the experimental mechanical event (channels 15 and 18). The channels activated in response to the social and mechanic events overlapped but were not identical. Hence, separate ROIs were computed for the social and mechanical events. The hemodynamic response curves for these two ROIs are displayed in Figure 4. One sample *t*-tests (one-tail) indicated that the mean response obtained in the ROI identified for the social event (*M* = 0.416, *SD* = 0.462) differed significantly from 0, *t*(17) = 4.57, *p* < 0.001, *d* = 1.078, BF = 233.84. Likewise, the mean response obtained in the ROI identified for the mechanical event (*M* = 0.458, *SD* = 0.570) also differed significantly from 0, *t*(15) = 3.21, *p* = 0.003, *d* = 0.802, BF = 17.00.

Channel level data for the control events were treated in the same manner as described for the experimental events and are reported in Supplementary Appendix A (HbO) and Supplementary Appendix B (HbR). To compare hemodynamic responses to the experimental versus control events we computed values for the ROIs identified in the experimental conditions. That is, for the control social event we averaged the responses obtained at channels 14, 15, and 18 and for the control mechanical event we averaged the responses obtained at channels 15 and 18. The variances obtained in response to the experimental and control social events were unequal (Levene’s test, *F* = 5.85, *df* = 1, *p* = 0.021). Hence, we used non-parametric statistics for comparison of the experimental and control data. A Mann–Whitney *U* test revealed that the infants who viewed the experimental social event had significantly greater activation in the superior temporal ROI (channels 14, 15, and 18) than the infants who viewed the social control event (*M* = 0.025, *SD* = 1.172), *U* = 223.00, *p* = 0.027, rank biserial correlation (effect size) = 0.377. One sample *t*-tests (one-tail) indicated that the mean response to the control social event did not differ significantly from 0, *t*(17) < 1. The variances obtained in response to the experimental and control mechanical events did not differ significantly (Levene’s test, *F* = 3.049, *df* = 1, *p* = 0.090) so parametric tests were used. Student’s *t*-test revealed that the activation observed in the superior temporal ROI (channels 15 and 18) differed significantly for the infants who viewed the experimental as compared to the control (*M* = −0.336, *SD* = 1.455) mechanical event, *t*(32) = 2.046, *p* = 0.025, *d* = 0.703. One sample *t*-tests (one-tail) indicated that the mean response to the control mechanical event did not differ significantly from 0, *t*(17) < 1.

Finally, significant activation was obtained in channel 7 in response to the control mechanical event (Supplementary Appendix A). This response differed significantly from that obtained in channel 7 to the experimental mechanical events, *t*(29) = −2.70, *p* = 0.011, suggesting an area in the left temporal cortex that is sensitive to dynamic mechanical entities not involved in interaction events. This was the only activation obtained in left hemisphere.

Hemodynamic responses functions for all channels in the right hemisphere, by event (social and mechanical) and condition (experimental and control) are displayed in Supplementary Appendix E.

#### Optical Imaging Data: Additional Analyses

The present experiment was designed to test cortical responses to two broad categories of events: social and mechanical. Although we did not expect the events within each category (i.e., help versus hinder and push versus pull) to generate different cortical responses, we felt compelled to test this expectation given recent electrophysiological data suggesting that viewing agents involved in helping as compared to hindering behavior elicits a larger P400 over posterior temporal areas (Cowell and Decety, 2015; Gredebäck et al., 2015). We were concerned primarily with events of the experimental condition. We tested responses to the help/hinder and pull/push events at the ROIs identified above. In the *experimental social condition*, the responses obtained in the right ROI to the help events (*M* = 0.450, *SD* = 0.541) and hinder events (*M* = 0.420, *SD* = 0.577) events did not differ significantly, *t*(16) < 1. In the *experimental mechanical condition*, right ROI responses to the push (*M* = 0. 557, *SD* = 1.149) and pull (*M* = 0.340, *SD* = 0.615) events did not differ significantly, *t*(15) < 1.

These analyses reveal that within each event category HbO responses did not differ by event type, suggesting that differences observed in the timing of electrophysiological responses are not evident in the location of hemodynamic responses. However, these results are based on fewer trials than the original analyses: infants saw only three trials of each event type, leading us to interpret these results with caution.

#### Behavioral Data: Additional Results

Distinct patterns of cortical activation were obtained in response to the social as compared to the mechanical events, suggesting these two types of events engaged different processing networks. If so, we would expect different behavioral responses to these two event types as well. To test this hypothesis, we used the choice task of Hamlin et al. (2007). Infants aged 7–8 months [*N* = 26, 14 males, age in day *M* = 240 (*SD* = 17); range = 217–266] saw one of the two experimental test events: social (*n* = 13) or mechanical (*n* = 13). Neuroimaging data were not collected. After viewing the three pairs of tests trials appropriate for the event condition to which they were assigned, infants were presented with a fourth pair of objects (Figure 2C) side-by-side, 20 cm apart (center to center), and centered on a 60 cm × 50 cm foam core board in front of the apparatus. Infant’s sat approximately 8 cm from the front edge of the foam core board. Half the infants saw the helper on the left, the other half saw the helper on the right. The foam core board was presented at midline directly within the infant’s reach. The experimenter, who was positioned directly across the foam core board, looked at the infant and asked “Hey baby, want to pick one?” The experimenter then looked down to prohibit social cuing. Once the baby touched an object the trial ended. Ten of the 13 infants (77%) who viewed the social events chose the helper. In contrast, six of the 13 infants (46%) who viewed the mechanical events chose the puller. The proportion of infants who chose the helper, but not the proportion who chose the pusher, differed significantly from chance, *p* < 0.05.

A separate group of infants aged 7–8 months [*N* = 13, 9 males, age in day *M* = 256 (*SD* = 20); range = 217–273] were tested using the same protocol except that they were shown the control social (*n* = 6) or mechanical (*n* = 7) events. For several reasons, including a lab move, we were unable to obtain a larger sample size for this experiment. Three of the six infants (50%) who viewed the control social events chose the “helper” and four of the 7 infants (57%) who viewed the mechanical events chose the “pusher.” Neither the proportion of infants who chose the helper, nor the proportion who chose the pusher, differed significantly from chance, *p* < 0.05. Give the small sample size, we interpret the control data with caution.

Two findings emerged. First, infants showed a preference for a social agent that engaged in cooperative as compared to uncooperative behavior; infants showed no preference for agents engaged in mechanical interactions. Second, when the social entities engaged in actions that were neutral (i.e., moved up or down a hill in synchrony), infants showed no preference for one social entity over another. These results provide converging evidence for the conclusion that infants distinguish between social and mechanical interactions and join a substantive body of research (for a meta-analysis see Margoni and Surian, 2018; for a review see Holvoet et al., 2016) demonstrating that infants’ preferences are reserved for entities that engage in prosocial behavior.

### Discussion of Experiment 1

As expected, viewing social interaction events elicited activation in the superior temporal region, although activation was obtained only in the right hemisphere. Unexpectedly, viewing mechanical interaction events also elicited activation in the right superior temporal region. Note however, that the ROI identified in response to the mechanical interaction events was overlapping with, but not identical to, the ROI identified in response to the social interaction events. Lloyd-Fox et al. (2009) also observed some right temporal activation to dynamic mechanical stimuli in 5-month-olds, but the activation was significantly less robust, in both the number of channels activated and the magnitude of the responses, than that observed in response to the social stimuli.

Why did we observe a robust response to both the social interaction *and* mechanical interaction event in the right superior temporal region? One important attribute the two events shared is that they both contained a causal structure. The action of one entity (the agent) caused the other entity (the patient) to move along a prescribed pathway toward an endpoint or goal. When the events lacked a causal structure, when the entities moved synchronously but did not interact, activation was not obtained in the right superior temporal region. One might question, then, whether the infants attended only to the causal structure and failed to perceive the difference in the ontological category to which the help/hinder (social) and push/pull (mechanical) interaction events belonged. The data from the behavioral task argue against this interpretation. After viewing social events, and when given a choice, infants showed a preference for the entity that acted in a cooperative as compared to non-cooperative way, a finding consistent with a large body of research (Hamlin et al., 2007, 2013; Hamlin and Wynn, 2012; Pun et al., 2018; Sommerville et al., 2018; for reviews see Holvoet et al., 2016; Margoni and Surian, 2018). In contrast, after viewing mechanical events, infants showed about equal preference for an object that pulled another object up a hill as compared to pushed another object down a hill. If infants perceived the events as ontologically distinct, one as social and the other as mechanical, why was this not evident in the cortical responses? One possibility is that cortical networks that respond to social as compared to mechanical interactions are largely overlapping at this age, an interpretation supported by the finding that the two event types (social and mechanical) resulted in distinct but overlapping ROIs. Another (not necessarily competing) possibility is that cortical areas that show the greatest specialization lie deeper in the brain, perhaps in inferotemporal areas, and hence not easily captured with fNIRS.

## Experiment 2

One of the goals of the current work was to assess the extent to which the specialization of cortical networks important for the processing of social and non-social physical entities changes during infancy. Hence, in Experiment 2 we tested infants aged 11–18 months using the procedure of Experiment 1. On the basis of previous work showing increased specialization in the ventral stream between 4 and 18 months (Wilcox et al., 2012, 2014), we expected that temporal regions might become more specialized for the processing of social and mechanical entities by the second year.

### Methods

#### Participants

Infants aged 11–18 months participated [*N* = 39; 17 females; age in days *M* = 475 (*SD* = 62.7) and range = 321–566 days]. Nineteen additional infants were tested but eliminated from the sample due to the infant removing the headgear (*n* = 4), fussiness or crying (*n* = 1), or difficulty obtaining an optical signal (*n* = 14). An additional 10 infants were not tested because they refused to put on the headgear. Infants were pseudo-randomly assigned to one of two conditions: experimental (*n* = 19) or control (*n* = 20).

Parents reported their infant’s ethnicity as Hispanic (*n* = 8), non-Hispanic (*n* = 30), or unknown/not reported (*n* = 1). Additionally, parents reported their infant’s race as White/Caucasian (*n* = 32), Black/African American (*n* = 1), multiple races (*n* = 4), or unknown/not reported (*n* = 2).

#### Task and Procedure

The task and procedure were identical to that of Experiment 1.

#### Instrumentation and Processing of fNIRS Data

Instrumentation and processing of fNIRS data were identical to that of Experiment 1. Mean head circumference did not differ significantly by condition (experimental *M* = 46.1, *SD* = 1.65 and control *M* = 47.4, *SD* = 1.62), *t* < *1*, *df* = 37. The head circumference of the infants tested ranged from 43 to 50 cm; the difference in the amount of skull covered by the left and right segments of the headgear, each, differed by 1.58 cm between the smallest and largest head circumference, less than the source-detector distance.

As in Experiment 1, in order to be included in the analysis, infants needed to complete at least 3 of the 6 trials for each event (social and mechanical). On the basis of the processing and looking time criteria, criteria 70 of 468 possible trials (15.0%) were eliminated from analysis. The percentage of trials eliminated did not vary significantly between events within each condition: experimental social, 18 of 114 trials (15.8%) as compared to experimental mechanical, 19 of 114 (16.7%) trials, *z*-score < 1; control social, 15 of 120 trials (12.5%) as compared to control mechanical, 18 of 120 trials (15%), *z*-score < 1.

Also as in Experiment 1, infants were required to contribute data from at least 13 of the total 20 channels, and at least 5 of 10 channels in a given hemisphere, for each of the two events (social and mechanical). Little’s (1988) Missing Completely at Random (MCAR) tests were conducted to assess whether the missing values were randomly distributed for each sample; each test was not significant, suggesting the data are MCAR (experimental, mechanical: χ_2_ = 104.969, DF = 112, *p* = 0.668; experimental, social: χ_2_ = 119.322, DF = 117, *p* = 0.423; control, mechanical: χ_2_ = 136.581, DF = 146, *p* = 0.700; control, social: χ_2_ = 133.276, DF = 149, *p* = 0.818). The overall percentage of channel-level missing data averaged across event and condition was 16.6%.

### Results

#### Preliminary Analyses

Preliminary analysis of the looking time and optical imaging data revealed no significant main effects or interactions involving sex.

#### Looking Time Data

Looking time data were analyzed as in Experiment 1. Mean looking times to experimental (*M* = 11.7, *SD* = 0.44) and control (*M* = 11.6 s, *SD* = 0.60 s) social events did not statistically differ, *t*(37) < 1. Mean looking times to the experimental (*M* = 11.6, *SD* = 0.43) and control (*M* = 11.4 s, *SD* = 0.68) mechanical events also did not statistically differ, *t*(37) < 1.

#### Optical Imaging Data

**FIGURE 5:**
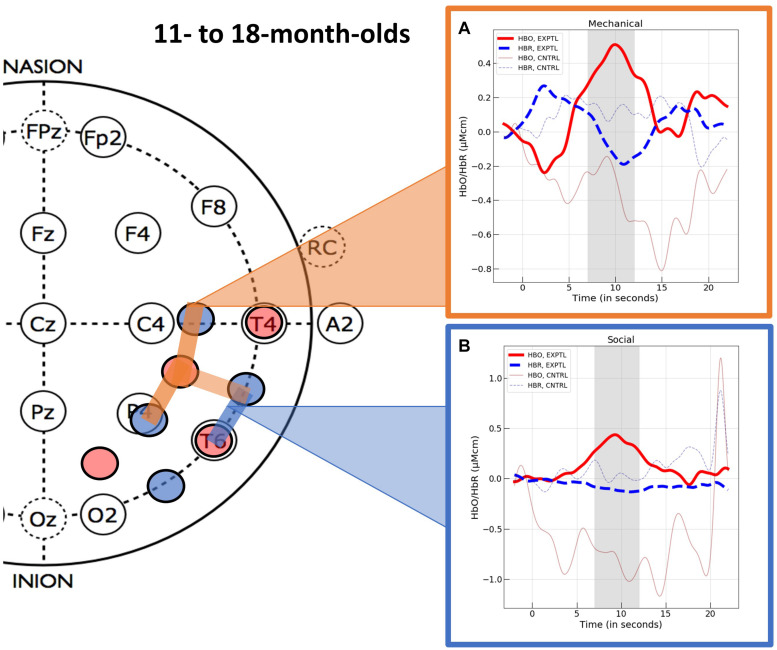
The hemodynamic response curves obtained in the ROIs identified in Experiment 2 with the 11- to- 18-month-olds in the **(A)** mechanical condition (the orange overlays) and the **(B)** social condition (the blue overlays). The time course of the curves (x-axis) includes 2 s prior to the test event, the 12 s test trial (highlighted gray is the 5 s time epoch over which we averaged), and the 10 s baseline event. Units on the y-axis are moles.

The optical imaging data were processed and analyzed as in Experiment 1. Mean hemodynamic responses obtained at each of the 20 channels (10 channels within each hemisphere) are reported in Supplementary Appendix C (HbO) and Supplementary Appendix D (HbR), by condition and event. First, we conducted channel-level analyses for the data obtained in the experimental condition. In response to the mechanical event, three spatially contiguous channels in the right superior temporal region (channels 15, 18, and 19) showed activation by passing all three criteria (*p* < 0.05, *d* > 5.0, and *BF* > 3). In response to the social event, one channel in the right hemisphere (channel 12) showed activation by passing all three criteria. The hemodynamic response curves are displayed in Figure 5.

Channel level data for the control events were treated in the same manner as described for the experimental events and are reported in Supplementary Appendix C (HbO) and Supplementary Appendix D (HbR). We then compared responses obtained to the experimental events with those obtained to the control events. For the experimental and control social events, we used the mean responses obtained at channel 12. The variances obtained in response to the experimental social and control social events at channel 12 were unequal (Levene’s test, *F* = 4.27, *df* = 1, *p* = 0.047). Hence, we used non-parametric statistics for comparison of the experimental and control social data. A Mann–Whitney *U* test revealed that the infants who viewed the experimental social event had significantly greater activation in the inferior temporal ROI (channel 12) than the infants who viewed the control social event (*M* = −0.834, *SD* = 1.996), *U* = 189.00, *p* = 0.012, *rank biserial correlation* (effect size) = 0.477. Finally, one sample *t*-tests (one-tail) indicated that the mean response to the control social event obtained at the inferior temporal ROI did not differ significantly from 0, *t*(15) = −1.672, *p* = 0.942. The variances obtained in response to the experimental and control mechanical events did not differ significantly (Levene’s test, *F* = 3.804, *df* = 1, *p* = 0.060) so parametric tests were used to compare responses across conditions. Student’s *t*-test revealed that the activation observed in the superior temporal mechanical ROI (channels 15, 18, and 19) differed significantly for the infants who viewed the experimental as compared to the control (*M* = −0.136, *SD* = 1.029) mechanical event, *t*(33) = 1.984, *p* = 0.028, *d* = 0.671. One sample *t*-tests (one-tail) indicated that the mean response to the control mechanical event at the superior temporal mechanical ROI, did not differ significantly from 0, *t*(17) ≤ 1.

Hemodynamic responses functions for all channels in the right hemisphere, by event (social and mechanical) and condition (experimental and control) are displayed in Supplementary Appendix F.

#### Optical Imaging Data: Additional Analyses

We compared responses obtained at the ROIs identified above within each event category. In the *experimental social condition*, the responses obtained in the right superior temporal ROI to the help (*M* = −0.053, *SD* = 0.744) and hinder (*M* = 0.085, *SD* = 0.895) events did not differ significantly, *t*(18) < 1. In the *experimental mechanical condition*, right superior temporal ROI responses to the push (*M* = 0.562, *SD* = 1.103) and pull (*M* = 0.440, *SD* = 0.923) events did not differ significantly, *t*(16) < 1.

In the *experimental social condition*, the responses obtained in the right inferior temporal area to the help (*M* = 0.310, *SD* = 0.486) and hinder (*M* = 0.412, *SD* = 0.993) events did not differ significantly, *t*(18) < 1. In the *experimental mechanical condition*, right inferior temporal area responses to the push (*M* = 0.071, *SD* = 0.421) and pull (*M* = 0.124, *SD* = 0.456) events did not differ significantly, *t*(13) < 1.

### Discussion of Experiment 2

Viewing mechanical interaction events elicited activation in the right superior temporal region, whereas viewing social interaction events elicited activation in a more inferior temporal region in the right hemisphere. The activation obtained in response to the experimental events differed significantly from that obtained in response to the control events. These results suggest that by the end of the first year, areas of temporal cortex are becoming specialized for processing of dynamic social and mechanical interaction events. No significant activation was obtained in these areas when the social and mechanical objects moved synchronously but did not interact, revealing that dynamic interaction, which contains a causal structure, is important to the patterns of activation observed.

## General Discussion

As predicted, the fNIRS results revealed a different pattern of activation in temporal cortex in response to the social as compared to the mechanical interaction events, in both age groups. Also as predicted, activation patterns were more specialized in the 11- to 18-month-olds than the 6- to 9-month-olds. This pattern of results was specific to interaction events and was not observed when the social or mechanical entities moved in synchrony but did not interact. This is one of the few fNIRS studies that has investigated age-related patterns of cortical activation and the first to provide insight into the functional development of networks specialized for processing of physical objects that move in accordance with basic mechanical laws, and social entities whose actions are guided by mental states.

### Early Emerging Processing Networks

Consist with previous research (Lloyd-Fox et al., 2009; Biondi et al., 2016; van der Kant et al., 2018), sensitivity to socially-relevant stimuli was obtained in the superior temporal cortex in the 6- to 9-month-olds of Experiment 1. One way the results of the current study differed from previous studies with this age group, however, was that we also found robust activation in the right superior temporal region in response to the mechanical interaction event. Why, in the current study, did we observe activation in the right superior temporal region to both the mechanical and social interaction events? One possible explanation, and one we suggested earlier, was that right temporal activation to both the social and the mechanical stimuli reflects sensitivity to the causal structure of agentive events. There is a large body of research showing that infants aged 6–9 months of age are sensitive to the causal structure of objects-in-motion events and can identify the causal agent within the context of these events (Leslie and Keeble, 1987; Saxe et al., 2007; Muentener and Carey, 2010; Schlottmann et al., 2012; for a review see Muentener and Bonawitz, 2017). Given the robust nature of these behavioral results, it is not surprising that this sensitivity would be instantiated in patterns of cortical activation. An alternative explanation for the activation pattern observed is that it reflects sensitivity to goal-directed behavior. By 5.5–6.5 months of age infants attribute goal-directed behavior to inanimate objects if the objects are self-propelled and follow paths that are efficient and consistent with the perceived goal (Luo and Baillargeon, 2005; Csibra, 2008). In the current study, the 6- to 9-month-old infants may have perceived the actions of both the social and mechanical entities as purposeful and goal directed. Of course, processing of the causal structure of events and of goal-directed behavior need not be mutually exclusive. It is not uncommon for events involving agents engaged in goal-directed behavior to also possess a causal structure and, in fact, it may be the presence of goal-directed behavior that leads infants to interpret an outcome as causal. Future studies will be needed to disentangle these two possible explanations for the pattern of fNIRS results obtained.

The current results also provide insight into a much larger network in temporal-frontal-parietal cortex that mediates processing of socially-relevant stimuli in young infants. Not only does the superior temporal cortex respond to the socially-relevant behavior of hands and faces (Lloyd-Fox et al., 2009; Biondi et al., 2016), but also to agentive, goal-directed behavior. We also know that temporal and/or frontal areas show specialized responses to the processing of faces, emotional vocalizations, and eye-gaze during the first 8 months (for reviews see Wilcox and Biondi, 2015a,b; Maria et al., 2018; McDonald and Perdue, 2018). And activation has been reported in the right parietal-temporal junction (PTJ) in 7-month-olds during processing of true and false belief events (Hyde et al., 2018). As we begin to put these pieces together, we will gain a better understanding of an early emerging social processing network.

### Greater Specialization of Processing Networks in Older Infants

A different pattern of cortical activation was observed with the 11- to 18-month-olds in Experiment 2: a right superior temporal region responded selectively to the mechanical interaction events, whereas a more inferior temporal region responded selectively to the social control events. These are the first data, of which we are aware, to show a difference in cortical responses to social and mechanical vignettes based on the nature of the interaction between the entities. Unlike many previous studies that have investigated the cortical basis of infant’s processing of social and mechanical entities, in these studies the perceptual characteristics of the objects and the way in which they moved was carefully controlled. For example, the mechanical and social entities had similar features (albeit configured differently) and moved along identical paths. Likewise, the primary difference between the experimental and control events was that the former included a cause for motion as a result of an interaction between the two entities involved in the event and the latter did not. Given carefully controlled stimuli, we can draw stronger interpretations about the basis for the hemodynamic responses observed.

Finally, it is important to place these results within a broader context. In the Section “Introduction,” we reported that the adult brain responds to meaningful categorical distinctions in the visual domain. For example, different patterns of cortical activation are obtained to biological as compared to non-biological motion, animate as compared to inanimate entities, and social as compared to mechanical interactions. However, there is another body of research that reports cortical sensitivity to similar types of distinctions within the auditory sensory domain (see Brefczynski-Lewis and Lewis, 2017 for a review). For example, neuroimaging and electrophysiological studies have revealed distinct cortical responses to human sounds (vocalizations and action-related sounds) as compared to mechanical (Engel et al., 2009; Lewis et al., 2011) and environmental (Engel et al., 2009; Lewis et al., 2011; Stavropoulos and Carver, 2016) sounds. Similar types of distinctions have been observed in infant studies. For example, distinct hemodynamic responses are obtained in response to voice as compared to non-voice stimuli (Lloyd-Fox et al., 2012), different electrophysiological responses are elicited by human as compared to environmental or mechanical sounds (Geangu et al., 2015), and infants are more likely to individuate objects on the basis of natural sounds as compared to non-natural sounds (Wilcox et al., 2006). These findings reveal just how important ontological distinctions are to human perception and cognition across the life span and sensory domains.

### Conclusion

Finally, the experiments reported here illustrate the feasibility of using fNIRS to study functional development of cortical structures. These findings add to a small but growing number of studies that have assessed infant’s processing of social (Lloyd-Fox et al., 2009, 2017) and non-social physical (Wilcox et al., 2012, 2014) entities during the first 2 years of life. Although there are a number of challenges associated with conducting studies that span a wide age range (e.g., older infants are less compliant, headgear must be adjustable) these types of studies are critical to our understanding of brain-behavior relations in the developing infant. In the future, implementation of a longitudinal design would provide better information about how patterns of functional activation change with time and experience.

## Data Availability Statement

The datasets generated for this study are available on request to the corresponding author.

## Ethics Statement

The studies involving human participants were reviewed and approved by IRB Texas A&M University and IRB Florida Atlantic University. Written informed consent to participate in this study was provided by the participants’ legal guardian/next of kin.

## Author Contributions

MB and AH contributed to study design, data collection, and data management. JS contributed to data processing, data manipulation, and manuscript preparation. TW contributed to study design, data analysis, and manuscript preparation. All authors contributed to the article and approved the submitted version.

## Conflict of Interest

MB is employed by the company Tobii. The remaining authors declare that the research was conducted in the absence of any commercial or financial relationships that could be construed as a potential conflict of interest.

## Footnotes

1 https://homer-fnirs.org/developers/

## Supplementary Material

Supplementary Appendix A Experiment 1 channel level HbO responses (Mean, *SD*) with associated statistics, of the infants who saw the experimental social and mechanical events **(top)** and the control social and mechanical events **(bottom)**. Channels 1–10 measure in left, and channels 11–20 measure in right, temporal areas. To be consider activated a channel had to meet three criteria: *p* < 0.05, *d* > 5.0, and BF > 3. Student’s *t*-test and BF analysis test the hypothesis that the response is >0. Shaded channels are those that were included in the analysis because they were activated for at least one of the experimental test events (social or mechanical). Spatially contiguous channels were averaged to form a single ROI.

Supplementary Appendix B Experiment 1 channel level HbR responses (Mean, *SD*) with associated statistics, of the infants who saw the experimental social and mechanical events **(top)** and the control social and mechanical events **(bottom)**. Channels 1–10 measure in left, and channels 11–20 measure in right, temporo-occipital areas. Student’s *t*-test and BF analysis test the hypothesis that the response is <0.

Supplementary Appendix C Experiment 2 channel level HbO responses (Mean, *SD*) with associated statistics, of the infants who saw the experimental social and mechanical events **(top)** and the control social and mechanical events **(bottom)**. Channels 1–10 measure in left, and channels 11–20 measure in right, temporo-occipital areas. To be consider activated a channel had to meet three criteria: *p* < 0.05, *d* > 5.0, and BF > 3. Student’s *t*-test and BF analysis test the hypothesis that the response is >0. Shaded channels are those that were included in the analysis because they were activated for at least one of the test events (social or mechanical). Spatially contiguous channels were averaged to form a single ROI.

Supplementary Appendix D Experiment 2 channel level HbR responses (Mean, *SD*) with associated statistics, of the infants who saw the experimental social and mechanical events **(top)** and the control social and mechanical events **(bottom)**. Channels 1–10 measure in left, and channels 11–20 measure in right, temporo-occipital areas. Student’s *t*-test and BF analysis test the hypothesis that the response is <0.

Supplementary Appendix E The hemodynamic response curves obtained for channel level HbO (red) and HbR (blue) responses in channels 11–20 in Experiment 1. The time course (*x*-axis) includes 2 s prior to the test event, the test trial (highlighted gray area is the 5 s time epoch over which we averaged), and the 10 s baseline event. Units on the *y*-axis are moles.

Supplementary Appendix F The hemodynamic response curves obtained for channel level HbO (red) and HbR (blue) responses in channels 11–20 in Experiment 2. The time course (*x*-axis) includes 2 s prior to the test event, the test trial (highlighted gray area is the 5 s time epoch over which we averaged), and the 10 s baseline event. Units on the *y*-axis are moles.
