# Supplementary material for: Cortical Activation to Social and Mechanical Stimuli in the Infant Brain
Source: Front Syst Neurosci. 2021 Jun 24;15:510030. doi: 10.3389/fnsys.2021.510030 (PMC8264292; doi:10.3389/fnsys.2021.510030)

Appendix A. Experiment 1 channel level HbO responses (Mean, SD) with associated statistics, of the infants who saw the experimental social and mechanical events (top) and the control social and mechanical events (bottom). Channels 1–10 measure in left, and channels 11–20 measure in right, temporal areas. To be consider activated a channel had to meet three criteria: $p < .05$, $d > 5.0$, and BF > 3. Student's *t*-test and BF analysis test the hypothesis that the response is > 0. Shaded channels are those that were included in the analysis because they were activated for at least one of the experimental test events (social or mechanical). Spatially contiguous channels were averaged to form a single ROI.

		**Experimental Mechanical**								**Experimental Social**							
	**Channel Name**	***Mean***	***SD***	***t-value***	***df***	***p-value (1-tail)***	***Cohen's d***	***BF***		***Mean***	***SD***	***t-value***	***df***	***p-value (1-tail)***	***Cohen's d***	***BF***	
	Channel 1	-0.036	0.786	-0.175	14	0.284	-0.045	0.232		0.077	0.498	0.635	16	0.1335	0.154	0.429	
	Channel 2	0.361	1.254	1.152	15	0.067	0.288	0.768		0.114	0.525	0.921	17	0.0925	0.217	0.566	
	Channel 3	0.233	0.935	0.996	15	0.0835	0.249	0.642		0.022	0.75	0.126	17	0.2255	0.03	0.268	
	Channel 4	0.116	0.483	0.957	15	0.0885	0.239	0.614		0.187	0.536	1.48	17	0.0395	0.349	1.118	
**Left Hemisphere**	Channel 5	0.179	0.574	1.248	15	0.058	0.312	0.861		0.267	0.595	1.905	17	0.0185	0.449	2.042	
	Channel 6	0.075	0.372	0.809	15	0.1075	0.202	0.524		0.106	0.61	0.739	17	0.1175	0.174	0.466	
	Channel 7	-0.179	1.178	-0.606	15	0.3615	-0.152	0.173		0.094	0.657	0.609	17	0.1375	0.144	0.409	
	Channel 8	0.131	0.521	0.973	14	0.087	0.251	0.64		0.266	0.838	1.308	16	0.0525	0.317	0.91	
	Channel 9	0.058	0.453	0.51	15	0.1545	0.127	0.39		0.226	0.695	1.379	17	0.0465	0.325	0.979	
	Channel 10	-0.358	0.856	-1.672	15	0.471	-0.418	0.11		-0.122	0.655	-0.791	17	0.39	-0.187	0.149	
	Channel 11	-0.128	0.563	-0.906	15	0.405	-0.227	0.149		-0.036	0.427	-0.361	17	0.3195	-0.085	0.19	
	Channel 12	0.146	0.639	0.883	14	0.098	0.228	0.58		-0.09	0.439	-0.817	15	0.3935	-0.204	0.155	
	Channel 13	-0.026	0.727	-0.141	15	0.2775	-0.035	0.231		-0.227	0.762	-1.263	17	0.444	-0.298	0.12	
	Channel 14	0.221	0.677	1.306	15	0.053	0.327	0.925		0.321	0.576	2.363	17	0.0075	0.557	4.195	*
**Right Hemisphere**	Channel 15	0.485	0.686	2.826	15	0.003	0.707	8.863	*	0.392	0.447	3.718	17	<0.001	0.876	47.044	*
	Channel 16	0.127	0.797	0.64	15	0.133	0.16	0.442		-0.095	0.697	-0.58	17	0.3575	-0.137	0.167	
	Channel 17	-0.177	0.376	-1.885	15	0.48	-0.471	0.104		0.086	0.524	0.697	17	0.124	0.164	0.446	
	Channel 18	0.431	0.573	3.008	15	0.002	0.752	12.042	*	0.578	0.598	4.099	17	<0.001	0.966	96.167	*
	Channel 19	0.192	0.409	1.881	15	0.02	0.47	2.006		0.279	0.691	1.714	17	0.026	0.404	1.544	
	Channel 20	-2.06E-04	1.111	-7.43E-04	15	0.25	-1.86E-04	0.255		0.176	0.671	1.113	17	0.0705	0.262	0.704	

		**Control Mechanical**								**Control Social**							
	**Channel Name**	***Mean***	***SD***	***t-value***	***df***	***p-value (1-tail)***	***Cohen's d***	***BF***		***Mean***	***SD***	***t-value***	***df***	***p-value (1-tail)***	***Cohen's d***	***BF***	
	Channel 1	0.342	1.755	0.728	13	0.12	0.195	0.507		0.164	1.435	0.428	13	0.169	0.114	0.382	
	Channel 2	0.066	1.77	0.129	11	0.225	0.037	0.317		-0.318	1.777	-0.62	11	0.363	-0.179	0.195	
	Channel 3	0.251	1.748	0.517	12	0.1535	0.143	0.426		-0.795	4.623	-0.62	12	0.3635	-0.172	0.188	
	Channel 4	-0.192	1.105	-0.717	16	0.379	-0.174	0.159		0.42	1.296	1.338	16	0.05	0.324	0.945	
**Left Hemisphere**	Channel 5	0.222	2.274	0.352	12	0.1825	0.098	0.368		0.284	1.92	0.533	12	0.151	0.148	0.432	
	Channel 6	0.279	1.38	0.807	15	0.108	0.202	0.523		0.599	1.299	1.786	14	0.024	0.461	1.771	
	Channel 7	1.08	1.412	2.961	14	0.0025	0.765	10.778	*	0.496	1.361	1.365	13	0.049	0.365	1.035	
	Channel 8	-0.004	0.65	-0.027	16	0.2555	-0.007	0.244		0.406	1.157	1.448	16	0.0415	0.351	1.089	
	Channel 9	0.23	0.907	1.075	17	0.0745	0.253	0.674		0.239	1.66	0.611	17	0.1375	0.144	0.41	
	Channel 10	0.591	1.913	1.31	17	0.052	0.309	0.896		-0.155	2.087	-0.307	16	0.309	-0.074	0.201	
	Channel 11	0.328	2.748	0.478	15	0.16	0.119	0.379		0.213	1.378	0.618	15	0.1365	0.154	0.432	
	Channel 12	-0.139	1.518	-0.342	13	0.3155	-0.091	0.214		0.348	1.539	0.877	14	0.099	0.226	0.577	
	Channel 13	0.166	1.856	0.358	15	0.1815	0.09	0.341		0.501	2.684	0.769	16	0.1135	0.187	0.491	
	Channel 14	0.266	1.092	0.944	14	0.0905	0.244	0.62		-0.439	1.189	-1.429	14	0.4565	-0.369	0.124	
**Right Hemisphere**	Channel 15	-0.561	2.018	-1.111	15	0.429	-0.278	0.136		0.607	1.8	1.348	15	0.0495	0.337	0.975	
	Channel 16	-0.703	2.297	-1.261	16	0.4435	-0.306	0.124		0.294	1.676	0.724	16	0.12	0.176	0.469	
	Channel 17	0.235	1.313	0.695	14	0.1245	0.179	0.478		0.266	1.872	0.55	14	0.1475	0.142	0.416	
	Channel 18	0.066	1.107	0.248	16	0.202	0.06	0.302		0.155	1.258	0.524	17	0.1515	0.124	0.377	
	Channel 19	-0.456	1.56	-1.206	16	0.4385	-0.292	0.127		0.146	1.635	0.369	16	0.1795	0.089	0.335	
	Channel 20	-0.636	1.651	-1.492	14	0.4605	-0.385	0.121		-0.127	1.522	-0.324	14	0.3125	-0.084	0.21	

Appendix B. Experiment 1 channel level HbR responses (Mean, SD) with associated statistics, of the infants who saw the experimental social and mechanical events (top) and the control social and mechanical events (bottom). Channels 1–10 measure in left, and channels 11–20 measure in right, temporo-occipital areas. Student's *t*-test and BF analysis test the hypothesis that the response is < 0.

		Experimental Mechanical							Experimental Social						
	**Channel Name**	***Mean***	***SD***	***t-value***	***df***	***p-value (1-tail)***	***Cohen's d***	***BF***	***Mean***	***SD***	***t-value***	***df***	***p-value (1-tail)***	***Cohen's d***	***BF***
	Channel 1	-0.042	0.402	-0.4	14	0.174	-0.103	0.3	-0.199	0.335	-2.445	16	0.0065	-0.593	0.166
	Channel 2	-0.045	0.234	-0.768	15	0.114	-0.192	0.133	-0.012	0.243	-0.214	17	0.2085	-0.05	0.14
	Channel 3	0.062	0.212	1.161	15	0.434	0.29	0.143	0.074	0.21	1.506	17	0.4625	0.355	0.222
	Channel 4	-0.041	0.237	-0.689	15	0.126	-0.172	0.145	-0.249	0.292	-3.615	17	0.0005	-0.852	0.111
**Left Hemisphere**	Channel 5	-0.151	0.572	-1.054	15	0.077	-0.264	0.128	-0.128	0.568	-0.953	17	0.0885	-0.225	0.097
	Channel 6	-0.093	0.404	-0.923	15	0.093	-0.231	0.156	-0.08	0.305	-1.111	17	0.0705	-0.262	0.153
	Channel 7	0.19	0.411	1.847	15	0.479	0.462	0.428	-0.066	0.328	-0.853	17	0.1015	-0.201	0.164
	Channel 8	-0.153	0.372	-1.595	14	0.033	-0.412	0.148	-0.153	0.389	-1.621	16	0.031	-0.393	0.122
	Channel 9	-0.209	0.531	-1.577	15	0.034	-0.394	0.183	-0.301	0.471	-2.709	17	0.0035	-0.639	0.115
	Channel 10	-0.072	0.552	-0.522	15	0.153	-0.131	1.495	-0.293	0.597	-2.081	17	0.013	-0.49	0.492
	Channel 11	0.124	0.176	2.821	15	0.497	0.705	0.581	0.055	0.209	1.117	17	0.43	0.263	0.326
	Channel 12	-0.019	0.423	-0.176	14	0.216	-0.045	0.155	0.006	0.407	0.063	15	0.2625	0.016	0.528
	Channel 13	-0.044	0.261	-0.668	15	0.129	-0.167	0.284	0.057	0.481	0.502	17	0.3445	0.118	0.845
	Channel 14	-0.002	0.246	-0.027	15	0.245	-0.007	0.125	-0.136	0.23	-2.508	17	0.0055	-0.591	0.086
**Right Hemisphere**	Channel 15	0.074	0.333	0.892	15	0.404	0.223	0.084	-0.049	0.335	-0.615	17	0.1365	-0.145	0.068
	Channel 16	-3.12E-04	0.233	-0.005	15	0.249	-0.001	0.17	0.032	0.271	0.507	17	0.3455	0.12	0.398
	Channel 17	0.076	0.283	1.074	15	0.425	0.268	2.016	0.114	0.237	2.05	17	0.486	0.483	0.156
	Channel 18	-0.023	0.303	-0.299	15	0.193	-0.075	0.082	-0.115	0.315	-1.548	17	0.035	-0.365	0.066
	Channel 19	-0.01	0.245	-0.158	15	0.219	-0.039	0.104	-0.135	0.246	-2.334	17	0.008	-0.55	0.102
	Channel 20	0.063	0.425	0.594	15	0.360	0.148	0.256	0.017	0.328	0.214	17	0.2915	0.05	0.128

		Control Mechanical							Control Social						
	**Channel Name**	***Mean***	***SD***	***t-value***	***df***	***p-value (1-tail)***	***Cohen's d***	***BF***	***Mean***	***SD***	***t-value***	***df***	***p-value (1-tail)***	***Cohen's d***	***BF***
	Channel 1	-0.174	1.316	-0.495	13	0.158	-0.132	0.405	-0.05	1.028	-0.18	13	0.215	-0.048	0.31
	Channel 2	0.114	0.426	0.925	11	0.407	0.267	0.168	0.303	1.017	1.034	11	0.419	0.298	0.16
	Channel 3	0.143	0.698	0.738	12	0.382	0.205	0.177	-0.163	0.847	-0.696	12	0.125	-0.193	0.505
	Channel 4	0.055	0.401	0.562	16	0.355	0.136	0.173	-0.207	0.762	-1.123	16	0.070	-0.272	0.727
**Left Hemisphere**	Channel 5	-0.276	0.678	-1.469	12	0.042	-0.407	1.2	-0.014	0.855	-0.058	12	0.239	-0.016	0.29
	Channel 6	0.275	0.825	1.332	15	0.450	0.333	0.124	-0.059	0.781	-0.292	14	0.194	-0.075	0.33
	Channel 7	0.309	0.752	1.591	14	0.467	0.411	0.117	0.162	0.865	0.699	13	0.376	0.187	0.175
	Channel 8	0.096	0.428	0.923	16	0.408	0.224	0.143	-0.168	0.704	-0.984	16	0.085	-0.239	0.62
	Channel 9	-0.112	0.698	-0.678	17	0.127	-0.16	0.438	-0.217	0.691	-1.333	17	0.050	-0.314	0.923
	Channel 10	0.004	0.772	0.02	17	0.254	0.005	0.239	-0.241	0.766	-1.295	16	0.054	-0.314	0.895
	Channel 11	-0.075	0.811	-0.368	15	0.180	-0.092	0.344	0.155	0.645	0.96	15	0.412	0.24	0.145
	Channel 12	0.274	0.702	1.459	13	0.458	0.39	0.127	0.034	0.879	0.148	14	0.279	0.038	0.236
	Channel 13	0.103	0.874	0.47	15	0.339	0.118	0.187	0.202	1.055	0.79	16	0.390	0.192	0.153
	Channel 14	0.063	0.785	0.309	14	0.310	0.08	0.212	-0.046	0.35	-0.504	14	0.156	-0.13	0.398
**Right Hemisphere**	Channel 15	0.102	0.813	0.501	15	0.344	0.125	0.184	0.047	0.962	0.195	15	0.288	0.049	0.223
	Channel 16	0.012	0.974	0.051	16	0.260	0.012	0.24	0.09	0.952	0.391	16	0.325	0.095	0.191
	Channel 17	-0.172	0.603	-1.104	14	0.072	-0.285	0.741	-0.151	0.641	-0.91	14	0.095	-0.235	0.597
	Channel 18	0.078	1.057	0.303	16	0.309	0.074	0.202	-0.337	0.782	-1.83	17	0.021	-0.431	1.825
	Channel 19	-0.132	1.204	-0.45	16	0.165	-0.109	0.361	-0.484	0.92	-2.169	16	0.012	-0.526	3.076
	Channel 20	-0.089	0.682	-0.507	14	0.155	-0.131	0.399	-0.43	1.25	-1.332	14	0.051	-0.344	0.974

Appendix C. Experiment 2 channel level HbO responses (Mean, SD) with associated statistics, of the infants who saw the experimental social and mechanical events (top) and the control social and mechanical events (bottom). Channels 1–10 measure in left, and channels 11–20 measure in right, temporo-occipital areas. To be consider activated a channel had to meet three criteria: $p < .05$, $d > 5.0$, and BF > 3. Student's *t*-test and BF analysis test the hypothesis that the response is > 0. Shaded channels are those that were included in the analysis because they were activated for at least one of the test events (social or mechanical). Spatially contiguous channels were averaged to form a single ROI.

		**Experimental Mechanical**								**Experimental Social**							
	**Channel Name**	***Mean***	***SD***	***t-value***	***df***	***p-value (1-tail)***	***Cohen's d***	***BF***		***Mean***	***SD***	***t-value***	***df***	***p-value (1-tail)***	***Cohen's d***	***BF***	
	Channel 1	0.032	0.434	0.286	14	0.195	0.074	0.329		0.077	0.799	0.409	17	0.172	0.096	0.34	
	Channel 2	0.327	0.787	1.66	15	0.030	0.415	1.47		0.289	1.013	1.243	18	0.058	0.285	0.809	
	Channel 3	0.109	0.589	0.739	15	0.118	0.185	0.488		-0.128	0.818	-0.662	17	0.371	-0.156	0.159	
	Channel 4	0.039	0.395	0.409	16	0.172	0.099	0.347		-0.085	0.587	-0.63	18	0.366	-0.144	0.158	
**Left Hemisphere**	Channel 5	0.081	0.874	0.372	15	0.179	0.093	0.345		0.066	0.651	0.444	18	0.166	0.102	0.343	
	Channel 6	0.324	0.975	1.328	15	0.051	0.332	0.951		-0.015	1.065	-0.058	17	0.262	-0.014	0.233	
	Channel 7	0.164	1.036	0.55	11	0.149	0.159	0.452		-0.566	2.25	-0.94	13	0.409	-0.251	0.156	
	Channel 8	-0.197	0.777	-1.047	16	0.423	-0.254	0.135		-0.094	0.635	-0.643	18	0.368	-0.148	0.157	
	Channel 9	-0.059	1.207	-0.195	15	0.288	-0.049	0.223		-0.074	0.782	-0.403	17	0.327	-0.095	0.185	
	Channel 10	0.397	1.181	1.212	12	0.062	0.336	0.88		-0.524	2.016	-1.008	14	0.418	-0.26	0.146	
	Channel 11	-0.085	0.47	-0.702	14	0.377	-0.181	0.169		-0.275	0.629	-1.853	17	0.480	-0.437	0.098	
	Channel 12	0.186	0.365	1.909	13	0.020	0.51	2.124		0.362	0.612	2.365	15	0.008	0.591	4.181	*
	Channel 13	0.132	0.426	1.162	13	0.067	0.311	0.811		0.122	0.568	0.856	15	0.102	0.214	0.551	
	Channel 14	0.177	0.446	1.64	16	0.030	0.398	1.411		0.007	0.794	0.036	18	0.243	0.008	0.244	
**Right Hemisphere**	Channel 15	0.408	0.696	2.272	14	0.010	0.587	3.604	*	-0.04	0.565	-0.298	17	0.308	-0.07	0.198	
	Channel 16	0.153	0.562	1.052	14	0.078	0.272	0.699		0.166	0.632	1.083	16	0.074	0.263	0.694	
	Channel 17	-0.336	0.656	-2.109	16	0.487	-0.512	0.094		0.045	0.594	0.327	18	0.187	0.075	0.309	
	Channel 18	0.413	0.599	2.844	16	0.003	0.69	9.352	*	-0.019	1.033	-0.081	18	0.266	-0.019	0.224	
	Channel 19	0.394	0.517	3.14	16	0.002	0.762	15.669	*	-0.11	0.336	-1.429	18	0.458	-0.328	0.11	
	Channel 20	-0.107	0.558	-0.792	16	0.390	-0.192	0.153		-0.183	0.43	-1.856	18	0.480	-0.426	0.095	

		**Control Mechanical**								**Control Social**							
	**Channel Name**	***Mean***	***SD***	***t-value***	***df***	***p-value (1-tail)***	***Cohen's d***	***BF***		***Mean***	***SD***	***t-value***	***df***	***p-value (1-tail)***	***Cohen's d***	***BF***	
	Channel 1	-0.152	0.729	-0.907	18	0.406	-0.208	0.137		0.103	0.947	0.45	16	0.165	0.109	0.361	
	Channel 2	-0.404	0.802	-2.136	17	0.488	-0.503	0.091		-0.215	1.071	-0.803	15	0.392	-0.201	0.156	
	Channel 3	-0.028	1.063	-0.102	14	0.270	-0.026	0.244		0.292	0.706	1.546	13	0.037	0.413	1.3	
	Channel 4	-0.145	0.515	-1.229	18	0.442	-0.282	0.119		-0.164	0.64	-1.056	16	0.424	-0.256	0.135	
**Left Hemisphere**	Channel 5	0.043	1.111	0.168	18	0.217	0.039	0.271		-8.00E-03	0.776	-0.041	16	0.258	-1.00E-02	0.242	
	Channel 6	-0.301	0.974	-1.273	16	0.445	-0.309	0.123		-0.172	1.11	-0.602	14	0.361	-0.155	0.179	
	Channel 7	0.208	0.928	0.866	14	0.100	0.224	0.57		0.023	1.205	0.071	13	0.236	0.019	0.285	
	Channel 8	0.115	0.45	1.113	18	0.070	0.255	0.691		-0.077	1.482	-0.214	16	0.292	-0.052	0.214	
	Channel 9	0.047	0.701	0.287	17	0.195	0.068	0.305		-0.074	0.717	-0.414	15	0.329	-0.104	0.193	
	Channel 10	0.22	0.761	1.192	16	0.063	0.289	0.789		-0.186	0.899	-0.829	15	0.395	-0.207	0.154	
	Channel 11	-0.111	1.044	-0.437	16	0.333	-0.106	0.186		-0.621	0.956	-2.599	15	0.495	-0.65	0.088	
	Channel 12	0.179	0.811	0.908	16	0.095	0.22	0.57		-0.834	1.996	-1.672	15	0.471	-0.418	0.11	
	Channel 13	-0.114	1.294	-0.383	18	0.324	-0.088	0.183		-0.235	0.865	-1.123	16	0.431	-0.272	0.131	
	Channel 14	-0.117	1.243	-0.387	16	0.324	-0.094	0.192		-0.234	1.096	-0.799	13	0.391	-0.214	0.166	
**Right Hemisphere**	Channel 15	-0.559	1.252	-1.786	15	0.477	-0.446	0.107		0.251	0.984	0.92	12	0.094	0.255	0.635	
	Channel 16	0.002	0.902	0.01	16	0.248	0.002	0.251		-0.242	0.982	-0.954	14	0.411	-0.246	0.15	
	Channel 17	0.038	0.873	0.178	16	0.215	0.043	0.286		0.234	1.112	0.815	14	0.107	0.21	0.54	
	Channel 18	-0.209	1.243	-0.694	16	0.376	-0.168	0.161		-0.106	1.19	-0.344	14	0.316	-0.089	0.208	
	Channel 19	0.143	0.932	0.616	15	0.137	0.154	0.432		0.457	1.302	1.312	13	0.053	0.351	0.97	
	Channel 20	0.186	0.722	0.999	14	0.084	0.258	0.659		0.404	0.979	1.544	13	0.037	0.413	1.298	

Appendix D. Experiment 2 channel level HbR responses (Mean, SD) with associated statistics, of the infants who saw the experimental social and mechanical events (top) and the control social and mechanical events (bottom). Channels 1–10 measure in left, and channels 11–20 measure in right, temporo-occipital areas. Student's *t*-test and BF analysis test the hypothesis that the response is < 0.

				Experimental Mechanical								Experimental Social				
	**Channel Name**	***Mean***	***SD***	***t-value***	***df***	***p-value (1-tail)***	***Cohen's d***	***BF***		***Mean***	***SD***	***t-value***	***df***	***p-value (1-tail)***	***Cohen's d***	***BF***
**Left Hemisphere**	Channel 1	0.056	0.296	0.737	14	0.382	0.19	0.166		0.095	0.572	0.706	17	0.378	0.166	0.156
	Channel 2	-0.054	0.221	-0.986	15	0.085	-0.246	0.635		0.007	0.679	0.042	18	0.259	0.01	0.23
	Channel 3	0.024	0.299	0.322	15	0.312	0.081	0.205		0.075	0.409	0.777	17	0.388	0.183	0.15
	Channel 4	0.024	0.239	0.41	16	0.328	0.099	0.189		0.042	0.381	0.475	18	0.340	0.109	0.173
	Channel 5	-0.022	0.615	-0.142	15	0.223	-0.035	0.285		0.142	0.676	0.918	18	0.408	0.211	0.136
	Channel 6	0.002	0.368	0.018	15	0.254	0.004	0.252		-0.045	0.655	-0.292	17	0.194	-0.069	0.307
	Channel 7	0.167	0.327	1.764	11	0.474	0.509	0.125		0.208	0.661	1.177	13	0.435	0.315	0.141
	Channel 8	0.075	0.571	0.542	16	0.351	0.132	0.175		0.101	0.4	1.1	18	0.429	0.252	0.125
	Channel 9	0.103	0.772	0.535	15	0.350	0.134	0.18		0.098	0.562	0.741	17	0.383	0.175	0.153
	Channel 10	-0.034	0.617	-0.2	12	0.211	-0.056	0.324		0.018	0.298	0.239	14	0.297	0.062	0.222
**Right Hemisphere**	Channel 11	0.021	0.169	0.476	14	0.340	0.123	0.192		-0.081	0.559	-0.613	17	0.137	-0.144	0.411
	Channel 12	0.039	0.35	0.415	13	0.329	0.111	0.205		-0.112	0.304	-1.476	15	0.040	-0.369	1.149
	Channel 13	0.018	0.178	0.38	13	0.323	0.101	0.209		-0.063	0.302	-0.839	15	0.104	-0.21	0.541
	Channel 14	-0.08	0.201	-1.632	16	0.031	-0.396	1.394		-0.084	0.69	-0.527	18	0.151	-0.121	0.37
	Channel 15	-0.054	0.335	-0.626	14	0.136	-0.162	0.447		-0.044	0.286	-0.649	17	0.132	-0.153	0.426
	Channel 16	0.155	0.636	0.947	14	0.410	0.245	0.15		-0.061	0.297	-0.843	16	0.103	-0.204	0.531
	Channel 17	0.068	0.228	1.223	16	0.440	0.297	0.126		0.128	0.272	2.054	18	0.487	0.471	0.09
	Channel 18	-0.117	0.358	-1.348	16	0.049	-0.327	0.957		-0.138	0.347	-1.733	18	0.025	-0.397	1.566
	Channel 19	-0.071	0.541	-0.543	16	0.149	-0.132	0.393		0.011	0.215	0.217	18	0.293	0.05	0.204
	Channel 20	0.132	0.313	1.736	16	0.475	0.421	0.105		0.134	0.185	3.16	18	0.499	0.725	0.071

				Control Mechanical								Control Social				
	**Channel Name**	***Mean***	***SD***	***t-value***	***df***	***p-value (1-tail)***	***Cohen's d***	***BF***		***Mean***	***SD***	***t-value***	***df***	***p-value (1-tail)***	***Cohen's d***	***BF***
**Left Hemisphere**	Channel 1	-0.045	0.355	-0.553	18	0.147	-0.127	0.379		0.149	0.472	1.301	16	0.447	0.316	0.122
	Channel 2	0.038	0.68	0.235	17	0.296	0.055	0.206		0.121	0.532	0.909	15	0.406	0.227	0.148
	Channel 3	0.058	0.528	0.427	14	0.331	0.11	0.197		0.099	0.432	0.857	13	0.399	0.229	0.162
	Channel 4	0.105	0.448	1.016	18	0.419	0.233	0.13		0.032	0.332	0.402	16	0.327	0.098	0.19
	Channel 5	-0.026	0.316	-0.365	18	0.180	-0.084	0.319		0.014	0.389	0.145	16	0.279	0.035	0.224
	Channel 6	-0.041	0.687	-0.246	16	0.203	-0.06	0.302		0.186	0.536	1.348	14	0.450	0.348	0.127
	Channel 7	0.155	1.027	0.583	14	0.358	0.151	0.18		0.263	0.607	1.622	13	0.468	0.434	0.12
	Channel 8	0.03	0.455	0.29	18	0.307	0.067	0.194		0.035	0.4	0.362	16	0.320	0.088	0.195
	Channel 9	0.006	0.474	0.051	17	0.260	0.012	0.234		-0.025	0.315	-0.318	15	0.189	-0.08	0.329
	Channel 10	-0.265	0.522	-2.095	16	0.013	-0.508	2.742		0.114	0.474	0.962	15	0.412	0.24	0.145
**Right Hemisphere**	Channel 11	0.106	0.614	0.709	16	0.378	0.172	0.159		0.141	0.437	1.291	15	0.446	0.323	0.126
	Channel 12	0.158	0.627	1.038	16	0.422	0.252	0.136		0.03	0.578	0.209	15	0.291	0.052	0.22
	Channel 13	-0.129	0.376	-1.495	18	0.038	-0.343	1.123		0.117	0.517	0.936	16	0.409	0.227	0.142
	Channel 14	-0.057	0.512	-0.459	16	0.163	-0.111	0.364		0.224	0.537	1.561	13	0.465	0.417	0.122
	Channel 15	0.184	0.737	0.999	15	0.417	0.25	0.142		0.018	0.74	0.09	12	0.268	0.025	0.261
	Channel 16	-0.093	0.319	-1.203	16	0.062	-0.292	0.8		-0.046	0.25	-0.707	14	0.123	-0.182	0.484
	Channel 17	-0.311	0.59	-2.175	16	0.011	-0.528	3.106		0.16	0.535	1.161	14	0.434	0.3	0.137
	Channel 18	-0.147	0.542	-1.119	16	0.070	-0.272	0.724		-0.07	0.419	-0.651	14	0.132	-0.168	0.458
	Channel 19	0.137	0.713	0.77	15	0.387	0.193	0.159		0.022	0.494	0.17	13	0.283	0.046	0.239
	Channel 20	-0.082	0.548	-0.58	14	0.143	-0.15	0.427		0.13	0.615	0.793	13	0.390	0.212	0.167

Appendix E. The hemodynamic response curves obtained for channel level HbO (red) and HbR (blue) responses in channels 11 – 20 in Experiment 1. The time course (x-axis) includes 2 s prior to the test event, the test trial (highlighted grey area is the 5 s time epoch over which we averaged), and the 10 s baseline event. Units on the y-axis are moles.

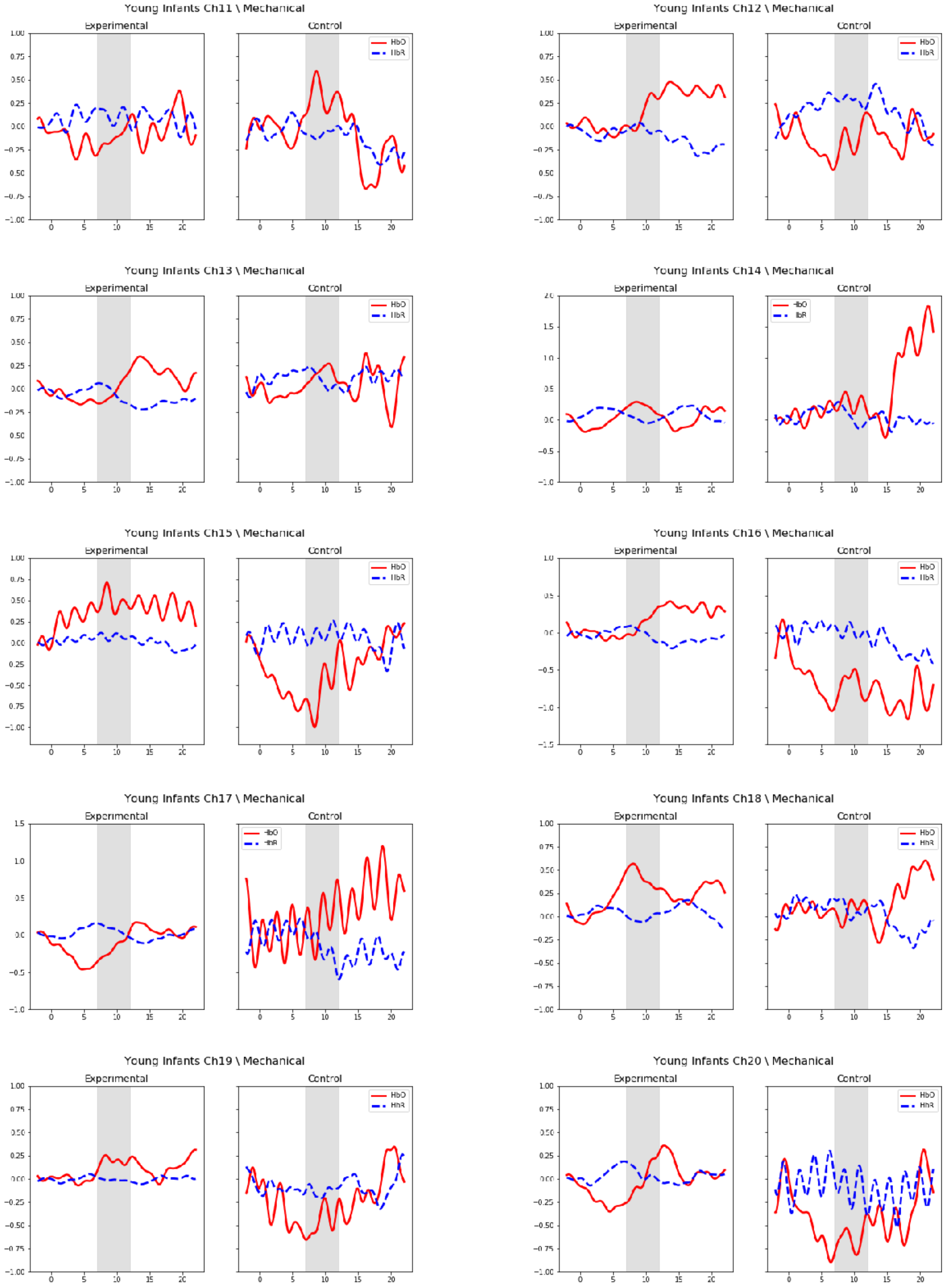

Appendix F. The hemodynamic response curves obtained for channel level HbO (red) and HbR (blue) responses in channels 11 – 20 in Experiment 2. The time course (x-axis) includes 2 s prior to the test event, the test trial (highlighted grey area is the 5 s time epoch over which we averaged), and the 10 s baseline event. Units on the y-axis are moles.

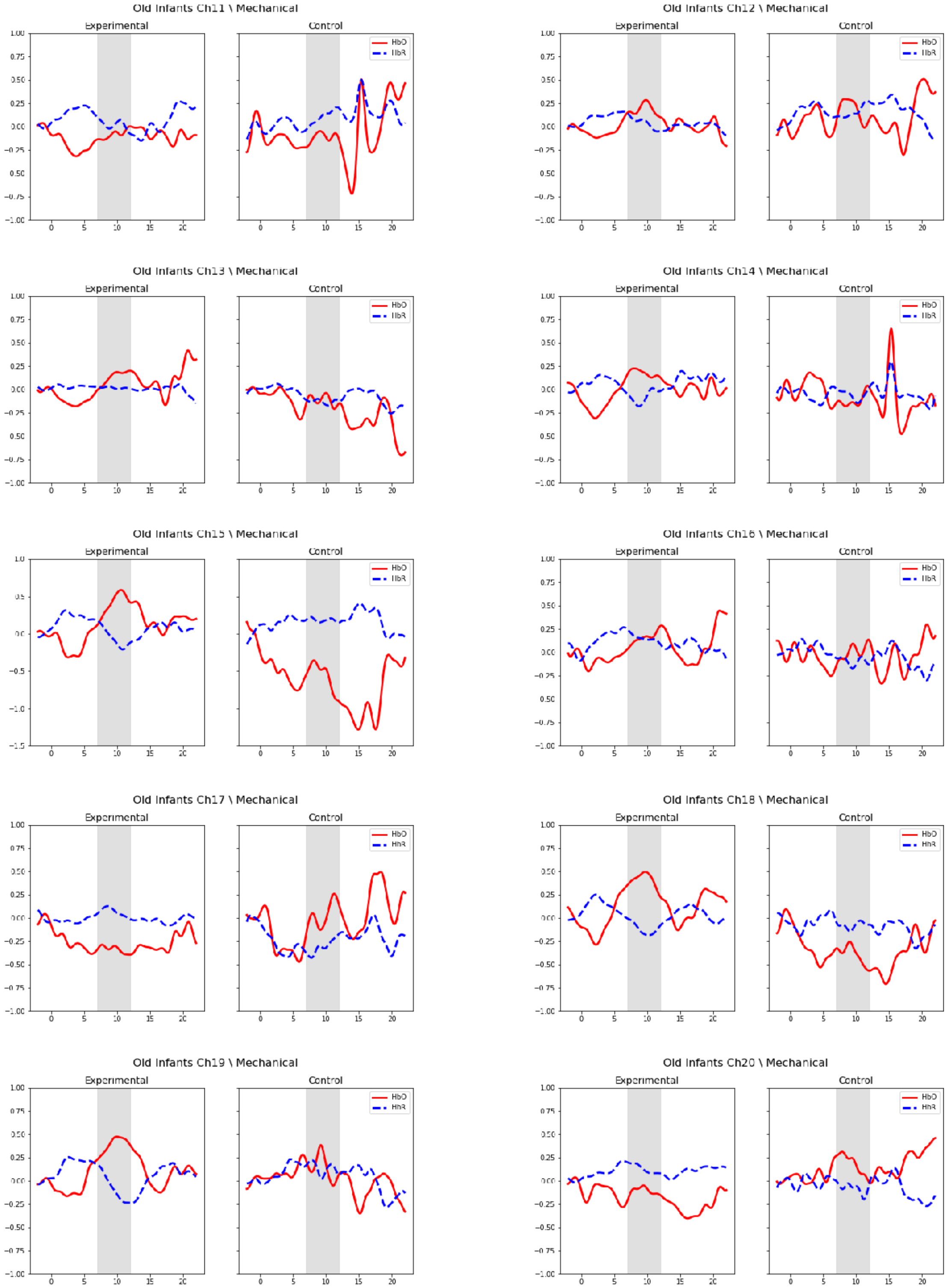

Supplement: Supplementary Appendix A — Experiment 1 channel level HbO responses (Mean, SD) with associated statistics, of the infants who saw the experimental social and mechanical events (top) and the control social and mechanical events (bottom). Channels 1–10 measure in left, and channels 11–20 measure in right, temporal areas. To be consider activated a channel had to meet three criteria: p < 0.05, d > 5.0, and BF > 3. Student’s t-test and BF analysis test the hypothesis that the response is >0. Shaded channels are those that were included in the analysis because they were activated for at least one of the experimental test events (social or mechanical). Spatially contiguous channels were averaged to form a single ROI. [file Data_Sheet_1.pdf]
